# Functional Reciprocity of Amyloids and Antimicrobial Peptides: Rethinking the Role of Supramolecular Assembly in Host Defense, Immune Activation, and Inflammation

**DOI:** 10.3389/fimmu.2020.01629

**Published:** 2020-07-31

**Authors:** Ernest Y. Lee, Yashes Srinivasan, Jaime de Anda, Lauren K. Nicastro, Çagla Tükel, Gerard C. L. Wong

**Affiliations:** ^1^Department of Bioengineering, University of California, Los Angeles, Los Angeles, CA, United States; ^2^UCLA-Caltech Medical Scientist Training Program, David Geffen School of Medicine, University of California, Los Angeles, Los Angeles, CA, United States; ^3^Department of Microbiology and Immunology, Lewis Katz School of Medicine, Temple University, Philadelphia, PA, United States; ^4^Department of Chemistry and Biochemistry, University of California, Los Angeles, Los Angeles, CA, United States; ^5^California Nano Systems Institute, University of California, Los Angeles, Los Angeles, CA, United States

**Keywords:** antimicrobial peptides, amyloids, self-assembly, Toll-like receptors, innate immunity, autoimmune diseases, neurodegenerative diseases

## Abstract

Pathological self-assembly is a concept that is classically associated with amyloids, such as amyloid-β (Aβ) in Alzheimer's disease and α-synuclein in Parkinson's disease. In prokaryotic organisms, amyloids are assembled extracellularly in a similar fashion to human amyloids. Pathogenicity of amyloids is attributed to their ability to transform into several distinct structural states that reflect their downstream biological consequences. While the oligomeric forms of amyloids are thought to be responsible for their cytotoxicity via membrane permeation, their fibrillar conformations are known to interact with the innate immune system to induce inflammation. Furthermore, both eukaryotic and prokaryotic amyloids can self-assemble into molecular chaperones to bind nucleic acids, enabling amplification of Toll-like receptor (TLR) signaling. Recent work has shown that antimicrobial peptides (AMPs) follow a strikingly similar paradigm. Previously, AMPs were thought of as peptides with the primary function of permeating microbial membranes. Consistent with this, many AMPs are facially amphiphilic and can facilitate membrane remodeling processes such as pore formation and fusion. We show that various AMPs and chemokines can also chaperone and organize immune ligands into amyloid-like ordered supramolecular structures that are geometrically optimized for binding to TLRs, thereby amplifying immune signaling. The ability of amphiphilic AMPs to self-assemble cooperatively into superhelical protofibrils that form structural scaffolds for the ordered presentation of immune ligands like DNA and dsRNA is central to inflammation. It is interesting to explore the notion that the assembly of AMP protofibrils may be analogous to that of amyloid aggregates. Coming full circle, recent work has suggested that Aβ and other amyloids also have AMP-like antimicrobial functions. The emerging perspective is one in which assembly affords a more finely calibrated system of recognition and response: the detection of single immune ligands, immune ligands bound to AMPs, and immune ligands spatially organized to varying degrees by AMPs, result in different immunologic outcomes. In this framework, not all ordered structures generated during multi-stepped AMP (or amyloid) assembly are pathological in origin. Supramolecular structures formed during this process serve as signatures to the innate immune system to orchestrate immune amplification in a proportional, situation-dependent manner.

## Introduction

Amyloids and antimicrobial peptides (AMPs) are two classes of proteins that have fascinating biophysical and structural properties. Until recently, they were thought to be distinct entities with vastly different functions. Amyloids were strictly pathologic and accumulation in tissues invariably led to diseases (1). In comparison, AMPs are considered essential components of the innate immune system, defending against invasive microbial infections and sounding the alarm to activate cellular-mediated immune responses (2, 3). Within the last 5–10 years, emerging work from collaborations between bioengineers, amyloid biologists, and immunologists has dramatically blurred the lines between amyloids and AMPs. AMPs and amyloids have strikingly similar structural and biophysical properties that enable them to self-assemble with immune ligands like DNA to amplify immune responses (4–6). Surprisingly, many amyloids possess hidden antimicrobial activity in addition to their known cytotoxic properties, suggesting a potential endogenous role in host defense (7, 8). AMPs and bacterial amyloids have also been implicated in the pathogenesis of autoimmune diseases like lupus and psoriasis (5, 9–13), parallel to the proinflammatory role of amyloids in neurodegeneration (14). Disentangling the molecular basis for the homeostatic and pathologic functions of both amyloids and AMPs has proven challenging (15).

**Figure 1:**
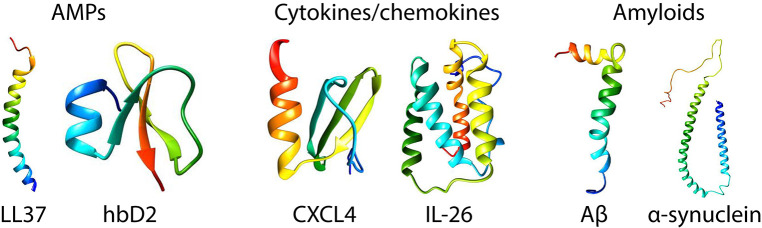
Structures of prototypical antimicrobial peptides, cytokines/chemokines, and amyloids. LL37 (22) and human β-defensin 2 (23) are canonical α-helical and β-sheet AMPs, respectively. CXCL4 (24) and IL-26 [homology model shown based on IL-19 (25)] are representative immune signaling molecules that also have known direct antimicrobial properties. Amyloid β (26) and α-synuclein (27) are the amyloids implicated in Alzheimer's disease and Parkinson's disease. The monomeric structures were taken from the Protein Data Bank (PDB) and visualized in Chimera (UCSF).

**Figure 2:**
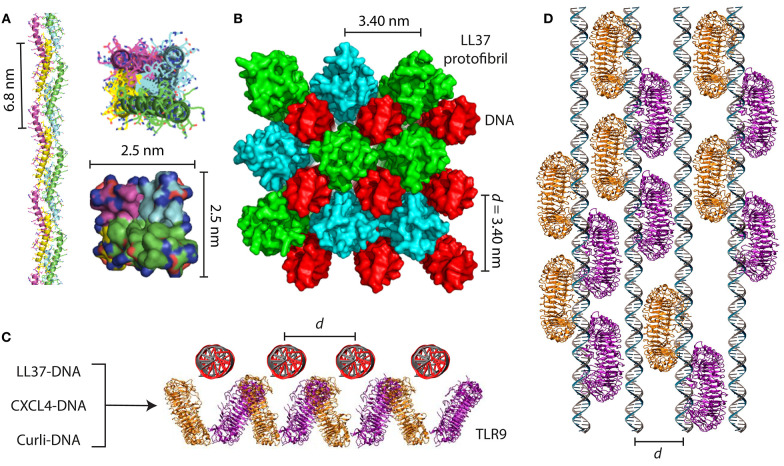
AMPs and amyloids organize immune ligands into spatially periodic nanocomplexes to amplify TLR activation. **(A)** LL37 self-assembles into a 4-fold amyloid-like superhelical protofibril in the presence of DNA. Hydrophobic residues are buried in the interior of the protofibril while cationic residues are exposed at the perimeter. **(B)** Structure of the LL37-DNA complex showing cross linking of spatially periodic DNA strands by LL37 protofibrils at an inter-DNA spacing of 3.40 nm, which is optimal for TLR9 binding and amplification of cytokine production. **(C)** End-on view and **(D)** top-down view of geometrically organized DNA immune complexes binding to clustered TLR9 in the endosomal membrane. In addition to the LL37-DNA complex, CXCL4-DNA complexes formed in scleroderma and curli-DNA complexes from *Salmonella* biofilms also demonstrate similar structural properties that enable amplification of TLR9 in immune cells and type I interferon production. **(A,B)** are adapted with permission from (4). **(C,D)** are adapted with permission from (44) and (10).

**Figure 3:**
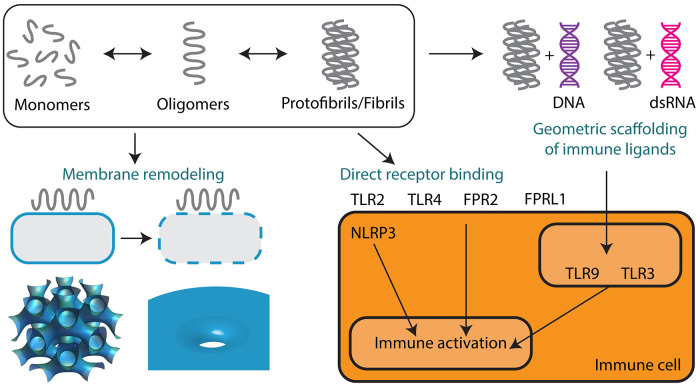
Supramolecular self-assembly of AMPs and amyloids enables membrane remodeling activity and immunomodulation. Monomers of AMPs and amyloids sequentially self-assemble into oligomers and protofibrils or fibrils. Oligomeric forms are predominantly responsible for mediating membrane permeation, including pore formation and membrane fusion leading to direct antimicrobial activity and cytotoxicity. Protofibrils and fibrils can signal to the innate immune system either by direct receptor binding or by the geometric scaffolding of immune ligands such as DNA and dsRNA. Both AMPs and amyloids can engage a broad range of immune receptors including TLR2, TLR3, TLR4, TLR9, FPR2, FPRL1, and NLRP3.

The goal of this review is to highlight fundamental studies that showcase the unexpected similarities between amyloid and AMP self-assembly and discuss how these findings can transform our understanding of their functional roles in host defense, inflammation, and disease. While some effort has been made in the literature to compare and contrast amyloids and AMPs, it has been difficult to identify common themes due to the sheer diversity of sequences and structures in both classes of molecules (Figure 1). Here, we begin by first providing a short overview of AMPs and their known antimicrobial and immunomodulatory functions. We focus on recent work from our group that outlines a novel emerging paradigm for understanding how AMPs talk to the innate immune system. We find that AMPs self-assemble into amyloid-like protofibrils that act as molecular templates to scaffold canonical immune ligands into spatially periodic nanocomplexes, which amplify immune responses via pattern-recognition receptors (PRRs) such as the Toll-like receptors (TLRs) (Figure 2). We demonstrate how this paradigm is general to other immune proteins beyond AMPs such as chemokines as well as other TLRs. We then discuss implications for the synergistic role of AMPs in normal host defense as well as in autoimmunity. In the second part of the review, we compare AMP self-assembly to amyloid self-assembly in the contexts of antimicrobial and membrane-remodeling activity (Figure 3). Lastly, we summarize how the functional similarities between AMPs and amyloids extends to bacterial amyloids as well in the realm of immunomodulation. By borrowing lessons and tools from the AMP literature, we find that amyloids potentially have endogenous functions beyond their pathologic consequences. We conclude by suggesting future research directions that can integrate our knowledge of AMP and amyloid biology to uncover mechanisms of disease and develop new targeted therapies.

## AMPs Organize Immune Ligands into Spatially Periodic Nanocomplexes to Amplify TLR Activation

AMPs are part of an ancient arm of the innate immune system that represents the first line of defense against microbial infections (2). AMPs are found in almost all living organisms including vertebrates, invertebrates, and plants (16–18), and can be broadly categorized by their secondary structures: the α-helical AMPs, β-sheet AMPs, AMPs with cross α-β structures, and extended linear peptides with specific enriched amino acids (19–21) (Figure 1). The prototypical human AMP is cathelicidin (LL37), which is an α-helical AMP with essential anti-infective and immunomodulatory functions (28, 29). Prototypical human β-sheet AMPs are the defensins. The mechanisms underpinning the antimicrobial activity of AMPs are thoroughly reviewed elsewhere but we briefly discuss it here (3, 30–32). In general, AMPs are cationic (+2 to +9) and amphiphilic with segregated groups of hydrophobic and polar/charged residues (2). These properties enable AMPs to electrostatically bind to negatively charged bacterial membranes and embed themselves into the membrane via hydrophobic interactions. Several models have been proposed for membrane permeation, including the “barrel-stave” model, “carpet” model, and “toroidal-pore” model (32). In the “barrel-stave” and “toroidal-pore” models, AMPs self-assemble into bundles that cylindrically insert into bacterial membranes to form aqueous pores, whereas in the “carpet” model, AMPs disintegrate the membrane via micellization (33). We have shown that AMP antimicrobial function correlates with its ability to induce negative Gaussian curvature (NGC) in bacterial membranes, a topological criterion for pore formation and membrane permeation (34). However, antimicrobial peptides are not only limited to membrane permeation. AMPs can also kill bacteria and fungi by disrupting metabolic gradients, inhibiting ribosomes, and binding to intracellular nucleic acids (35). However, the most underappreciated aspect of AMP function is their ability to amplify immune responses by autocrine signaling via PRRs such as TLRs. AMPs can signal through PRRs via direct binding. LL37 has been shown to be a chemoattractant for leukocytes by binding to the formyl peptide receptor-like 1 (FPRL1) (36). Furthermore, β-defensins are known to be chemotactic for monocytes and macrophages by binding to the CCR6 receptor (37), and β-defensin 2 is a known ligand for Toll-like receptor 4 (TLR4) (38). Despite this work, it was not known until recently whether AMPs could signal to PRRs without being direct ligands, or whether they could serve as chaperones by binding to immune ligands such as nucleic acids.

In a series of groundbreaking studies, Lande et al. showed that LL37 can break immune tolerance to self-DNA in diseases like lupus and psoriasis by forming insoluble complexes that are phagocytosed by immune cells. In these diseases, LL37 is overexpressed in the skin and blood and are predominantly produced by neutrophils and keratinocytes (39–41). LL37-DNA complexes are formed extracellularly and are internalized into the endosomes of plasmacytoid dendritic cells (pDCs), amplifying type I interferon (IFN-α) production by binding to Toll-like receptor 9 (TLR9). They also showed that other cationic AMPs in the skin possess a similar property, including the β-defensins and lysozyme (42). To understand the molecular basis for how LL37 and other AMPs signal through TLR9, we characterized the structures of numerous AMP-DNA complexes using X-ray scattering and correlated them with their ability to activate pDCs via TLR9 (43). We found that LL37 and β-defensins electrostatically self-assemble with DNA into spatially periodic grill-like nanostructures with well-defined inter-DNA spacings, and that the inter-DNA spacing within these complexes correlated directly with the quantitative degree of cytokine production (Figures 2A–D). The biophysics of the hierarchical electrostatic self-assembly of rigid polyelectrolytes like DNA has been well-described in the literature and is thoroughly discussed elsewhere (45–47). AMP-DNA complexes with spacings well-matched with the steric size of TLR9 enabled multivalent binding to clustered TLR9 on the endosomal membrane and IFN-α production orders of magnitude higher than expected from individual ligands (45). Surprisingly, this phenomenon was independent of the degree of endosomal uptake, suggesting that this differential response was solely due to differences in the nanostructures of the complexes. This conceptual transformation suggested that a much broader range of molecules could be predicted to activate TLR9 if they had the right physicochemical properties to organize and present DNA at optimal periodic positions that promote multivalent interactions with an ensemble of TLR9.

Inspired by this, we set out to discover general rules for how α-helical AMPs like LL37 can self-assemble into molecular templates for DNA binding and amplify immune responses. Previous work has shown that artificial patchy amphiphiles can be designed to self-assemble into various unique structures (48, 49). By combining computer simulations with X-ray structural characterization, we found that LL37 oligomerizes into a superhelical amyloid-like protofibril in the presence of DNA, with hydrophobic residues buried in the interior and outward-facing cationic residues (4) (Figures 2A,B). The LL37 protofibril cross-links DNA into a 4-fold coordinated lattice with inter-DNA spacings commensurate with the size of TLR9. We conducted experiments with other α-helical AMPs with different charge densities and hydrophobicities such as melittin (50) and buforin (51). We discovered that formation of this amyloid-like protofibril requires sufficient hydrophobicity to enable polymerization into a superhelix and cationic charge density well-matched to the high anionic charge density of DNA. Remarkably, we discovered that although melittin was able to form optimized complexes with DNA for TLR9 activation, its cytotoxicity to immune cells prevented cytokine production. By attenuating its cytolytic activity while retaining its ability to self-assemble into 4-fold coordinated nanocomplexes with DNA at the optimal inter-DNA spacing, we rescued its ability to activate TLR9 (4). This highlighted that there exist natural tradeoffs in antimicrobial and immunomodulatory functions of AMPs, and that we can deterministically modulate them by altering the AMP's physicochemical properties.

The next natural question to ask is whether this phenomenon is general to other immune ligands and innate immune receptors. Gallo and colleagues have previously shown that LL37 can break immune tolerance to double-stranded RNA (dsRNA) released from keratinocytes in psoriasis and other cutaneous diseases (52–55). Given the structural homology of TLR9 to Toll-like receptor 3 (TLR3) and DNA to dsRNA, respectively, we decided to map out the structural rules for immune activation of TLR3 by dsRNA complexes (56). We characterized the structures of numerous AMP-dsRNA complexes (LL37 and various truncated variants) and tested their ability to induce IL-6 production from psoriatic keratinocytes via TLR3. Cognate to LL37-DNA complexes, we found that LL37-dsRNA complexes formed nanocrystalline structures with well-defined inter-dsRNA spacings, and that complexes that maximally activated TLR3 had spacings perfectly matched with the steric size of TLR3. A mathematical model and computer simulation of TLR3 binding to spatially periodic AMP-dsRNA complexes recapitulated the experimental data and showed that both the inter-dsRNA spacing and the number of repeat units within the complexes were primary determinants of immune activation (56). This validated the idea that innate immune receptors like TLR9 and TLR3 can recognize both single ligands, as well as the crystallinity of spatially periodic, geometrically patterned ligands templated by molecular chaperones like AMPs.

As it turns out, this phenomenon is not limited to AMPs, but is rather general to other immune signaling proteins. Chemokines are a well-studied class of immune signaling molecules that are known to exert their biological activities by binding to G-protein coupled receptors (GPCRs) on the surface of immune cells. We discovered an unexpected signaling pathway for chemokine (C-X-C motif) ligand 4 (CXCL4)/platelet factor 4 (PF4) and its role in the pathogenesis of scleroderma. Interestingly, CXCL4 naturally self-assembles into an oligomeric homotetramer and has a cationic, amphipathic cross α-β structure that is homologous to that of defensin antimicrobial peptides (57) (Figure 1). It has also previously been shown to exert antimicrobial activity (58–62). CXCL4 is typically highly expressed in platelets and plays a key role in hemostasis and wound healing (63). CXCL4 is known to bind to anionic heparin, particularly in the context of heparin-induced thrombocytopenia (64–66), but its causal role in inflammatory diseases was unclear. We discovered that like LL37 and other AMPs, CXCL4 can self-assemble with microbial and self-DNA to form nanocomplexes to amplify IFN-α production via TLR9 within skin pDCs (Figures 2C,D). We identified CXCL4-DNA complexes in the blood and skin of scleroderma patients, and levels of these complexes correlated directly with the type I interferon signature (44). Surprisingly, this activity was independent of the canonical CXCL4 receptor, CXCR3. We predict that many other chemokines likely possess similar properties, since they share a structural backbone and have close physicochemical similarity, including the ability to self-assemble into oligomers. Taken together, our findings are consistent with a robust emerging conceptual framework where diverse classes of molecules can signal to the innate immune system by scaffolding endogenous immune ligands into spatially periodic nanocomplexes, rather than being direct agonists.

## Synergy Between the Antimicrobial and Immunomodulatory Properties of AMPs and Chemokines

Thus far, we have demonstrated that AMPs and chemokines are multifunctional, and can exert direct antimicrobial activity and modulate immune responses via PRRs. Due to their cationicity and amphipathicity, AMPs are capable of directly killing microbes through membrane permeation, inhibition of metabolic machinery, and disruption of electrostatic gradients. However, the same physicochemical features allow them to also self-assemble into ordered nanocrystalline complexes with immune ligands such as DNA and dsRNA by functioning as structural scaffolds. These complexes can potently induce inflammation by amplifying Toll-like receptor activation via receptor clustering, and the crystallinity of these complexes can determine the degree of immune amplification (43). What are the consequences of this multifunctionality for host defense?

Synergy between the dual antimicrobial and immunomodulatory functions of many AMPs and chemokines enables them to play an important role in protection against infections and in mediating autoimmune disease and inflammation. Certain AMPs and chemokines are capable of lysing and killing bacteria and presenting fragments of bacteria such as DNA to innate immune receptors. For instance, Meller et al. demonstrated that interleukin 26 (IL-26), a cytokine secreted by human interleukin-17 producing helper T cells (T_H_17), both kills bacteria and promotes immune sensing of bacterial and host cell death, driving the potent antimicrobial and proinflammatory function of T_H_17 cells (67) (Figure 1). IL-26 is a highly cationic and amphipathic protein that possess broad-spectrum antimicrobial activity against several gram-negative bacterial strains including *P. aeruginosa, E. coli*, and *K. pneumoniae*, and gram-positive bacteria *S. aureus* (67, 68). IL-26, like AMPs, can oligomerize into multimers and lyse bacteria by forming pores in their membranes. The antimicrobial properties of T_H_17 cell-derived IL-26 helps explain why patients defective in T_H_17 cells are highly susceptible to *S. aureus* infections (69), and why depletion of T_H_17 cells during infection by simian immunodeficiency virus results in the dispersal of gut bacteria (70).

Upon bacterial killing, T_H_17 cell-derived IL-26 triggers potent immune activation. IL-26 forms nanocrystalline complexes with bacterial DNA released during the antimicrobial response. These complexes are internalized into the endosomal compartments of pDCs and induce an amplified production of IFN-α via recruitment and super-selective binding of TLR9 receptors. Type I interferons are responsible for driving many proinflammatory responses, including CD8+ T cell activation (71, 72), T_H_1 cell differentiation (72), NK cell activation, dendritic cell maturation (73, 74), and promotion of antibody-secreting plasma cells (75). Consequently, their production has been shown to be beneficial in the context of extracellular bacterial infections, including the resolution and control of infections caused by *P. aeruginosa, S. pneumoniae*, and *E. coli* (76, 77), and reducing inflammation in mouse models of bacterial sepsis (78). In addition to serving as a direct antimicrobial, IL-26 has evolved the ability to amplify and regulate innate and adaptive responses to extracellular bacteria. Its dual functionality allows our immune system to more effectively clear bacterial infections. Modulating the endogenous activity of IL-26 may offer promising strategies to enhance our natural host defense against microbes.

IL-26 and CXCL4 are likely several of many examples of multifunctional molecules that play a synergistic role in host defense against microbes via direct killing and immunomodulation, in addition to their other homeostatic functions. Recently, other interferons like IFN-β (79) and IFN-γ (80) were shown to exhibit direct antimicrobial properties in addition to their known immunomodulatory functions. These findings suggest that the nature has evolved a way to bioconjugate multiple distinct functions into the same amino acid sequence (81), and that understanding how the immune system works requires us to examine these hidden functions.

## Comparison of AMP and Amyloid Self-Assembly

Here, we draw comparisons between the self-assembly of AMPs and the classical self-assembly of amyloids. Amyloids constitute a broad class of proteins that have the unique ability to aggregate into fibrils with characteristic secondary structures. The structural, physicochemical, and biological properties of AMPs are similar to those of many amyloid proteins. The majority of amyloids have a β-sheet secondary structure, but recently a subset of α-helical amyloids was identified (82, 83). Amyloids can be broadly categorized into those of eukaryotic and prokaryotic origins. Human endogenous amyloids are associated with over 50 distinct disease processes, the most famous of which is amyloid β-peptide (Aβ) in Alzheimer's disease (AD) (Figure 1). More and more proteins are being discovered to have amyloidogenic properties. Whether amyloids play a causal role in disease or are merely a consequence of disease is hotly debated. However, amyloids have unequivocally been shown to exhibit direct cytotoxic activity against human cells. The best data is available for Aβ, but many other amyloids have been shown to self-assemble into structures that can disrupt membranes (84) and signal to the immune system (Figure 3).

Aβ is the main component of amyloid plaques found within neurons in AD brains and is thought to induce cytotoxicity leading to neuronal cell death (85) via multiple mechanisms (86–89). Traditionally, Aβ has been characterized as a functional catabolic byproduct of amyloid precursor protein (APP) without much evidence for a possible endogenous homeostatic function (90). However, recent *in vitro* studies have shown that Aβ can exhibit AMP-like direct antimicrobial activity by disrupting membranes (91) and may play a role as an effector molecule of innate immunity, exhibiting broad-spectrum activity against several common and clinically relevant organisms (92) (Figure 3). In a directly related study, Kumar et al. highlighted the potent antimicrobial activity of Aβ and demonstrated its biological relevance in host defense through *in vivo* models of infection. Aβ expression is associated with increased host survival in both nematode and mouse models of bacterial (93) and viral infection (94). Low Aβ expression resulted in greater death of APP-KO mice after infection. The protective role of Aβ can be attributed to classic AMP mechanisms characterized by reduced microbial adhesion, bacterial membrane disruption, and entrapment of microbes by Aβ fibrils (93). Alternatively, low levels of fibrillar Aβ may signal to the immune system and elicit inflammation to keep the immune system or the infection in check. Low levels of Aβ can get cleared without amyloid deposition. Nonetheless, these data imply that Aβ possesses a normally protective role in host defense that, when dysregulated, can lead to neurodegenerative disease. Aβ may normally function as an endogenous inducible AMP that is cleared upon resolution of inflammation. However, when dysregulated in the right of genetic or environmental context, Aβ instead forms toxic amyloid oligomers leading to neuronal cell death and eventually deposits leading to chronic inflammation (95).

It is important to note that genetic factors may also be involved in the dysregulation of Aβ production in addition to environmental factors like bacterial and viral infections (96, 97). Overexpression of APP on chromosome 21 is associated with AD, and individuals with Down syndrome (Trisomy 21) are at a higher risk of AD relative to the population (96). In addition, Aβ from individuals with the “Arctic” mutation (E693G 669 on APP) tends to self-assemble into protofibrils at a much higher rate than the wild type protein (98). A larger number of additional genetic polymorphisms have been identified which affect Aβ cytotoxicity (99), but their consequences on Aβ in host defense is currently unknown. It is also possible that genetic polymorphisms in other immune and inflammatory genes can alter Aβ production and contribute to AD. For example, the apolipoprotein gene ApoE4 is another major genetic risk factor for AD (100, 101), and deficient clearance of Aβ is associated with disease (102). Further work will be required to elucidate how these genetic changes affect the function of Aβ in host defense and inflammation.

**Figure 4:**
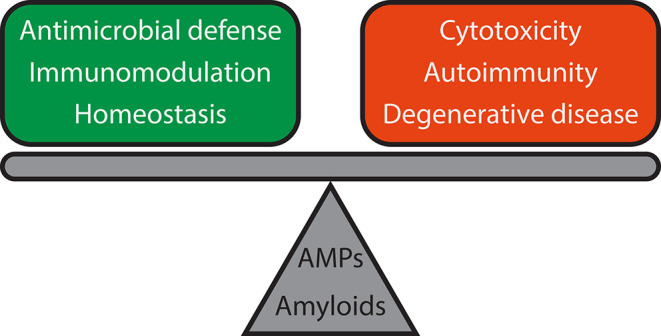
Functional reciprocity of AMPs and amyloids and consequences for host defense and immune signaling. Both AMPs and amyloids are involved in antimicrobial defense, immunomodulation, and homeostasis, but dysregulation can lead to adverse outcomes such as cytotoxicity, chronic inflammation, and autoimmune diseases like lupus, and degenerative diseases like Alzheimer's disease and Parkinson's disease. Further work will be required to elucidate the mechanisms of how such a delicate balance is attained.

Similarly, while AMPs are typically protective, dysregulation of AMP expression can lead to host cell toxicity, degenerative pathologies, and chronic inflammation and autoimmunity as described above (103–105) (Figure 4). For example, LL37 is a human cathelicidin AMP essential for normal immune function and protection against lethal infections (106). However, at elevated physiological concentrations, it is cytotoxic to host smooth muscle cells (107) and implicated in the pathogenesis of late-stage diseases including atherosclerosis, rheumatoid arthritis, and systemic lupus erythematosus (29). Interestingly, certain AMPs are deposited as amyloids in common human amyloidopathies including isolated atrial and senile seminal vesicle amyloidosis (7, 92, 108). In fact, a large number of naturally occurring AMPs including LL37 (4, 109), lysozyme (110), protegrin-1 (111), plant defensins (112), temporins (113, 114), histatin 5 (115), HAL-2 (116), uperin 3.5 (117), dermaseptin S9 (118), Cn-AMP2 (119), and longipin (120) and apolipoprotein A-I (121) from invertebrates form amyloids or amyloid-like fibrils *in vitro* and *in vivo*. A number of synthetic amyloid-inspired peptides have been designed primarily as novel broad-spectrum antibiotics (83, 122), and many AMPs are known to oligomerize before or upon membrane binding and pore formation (123, 124).

The potential protective effects of host-generated amyloids have only recently emerged (7, 8, 125) despite recognition of the association between chronic bacterial infections and amyloidosis for nearly a century (1). Findings related to the role Aβ plays in neuronal innate immune defense may extend to proteins associated with amyloidopathies other than AD, several of which have been shown to exhibit antimicrobial activity (18, 108, 126–129). Pathways that regulate innate immunity in AD and other amyloidopathies may serve as novel targets for therapeutic intervention. Parkinson's disease (PD)-associated α-synuclein has been long-studied as a model system for amyloid-mediated cytotoxicity (130–134) due to its propensity for membrane interactions (135, 136) via its N-terminal helix (137, 138) (Figure 1). Recently, it was shown to be antimicrobial against a variety of bacteria and fungi (139). Unexpectedly, it was found to be also involved in the chemoattraction of immune cells, suggesting a potential endogenous role in host defense (140). In human patients with chronic gut inflammation, α-synuclein was found to be upregulated in enteric neurons (141), a fascinating finding given that PD often begins in the gut as constipation before neurologic symptoms appear (142, 143). Disruption of the ability of α-synuclein to self-assemble into oligomers on neuronal membranes appears to be a potential therapeutic strategy in a nematode model of PD (144). Beyond Aβ and α-synuclein, several other amyloids or their fragments have been shown to have antimicrobial or membrane-lytic properties, including tau (145), islet amyloid polypeptide (IAPP) (146–148), human prion protein (128), superoxide dismutase (127), and endostatin (149). The functional bacterial amyloid curli, which is a key stromal component of *Salmonella* biofilms (150), was also shown to form cytotoxic oligomeric intermediates (151).

Interestingly, a recent machine learning tool originally trained to identify antimicrobial activity in α-helical AMPs identified a subset of naturally occurring amyloid peptides that possess predicted membrane-permeating activity (33, 152–154), among numerous other classes of molecules (155, 156). This demonstrates that data-driven approaches may be helpful in further identifying amyloids that are involved in host defense, but it is clear that much more work needs to be done to validate the extent and relevance of that function.

## Immunomodulatory Aspects of Amyloids and Similarity to AMPs

The functional similarities between AMPs and Aβ amyloids extend to bacterial amyloids as well. In bacterial biofilms, bacterial amyloids form the building blocks of the biofilm extracellular matrix alongside extracellular DNA (eDNA) (157). In a series of landmark papers, Tükel and colleagues showed that the biofilm amyloid curli from *Salmonella* and *E. coli* activated TLR2 (158–160) (Figure 3). Subsequent studies have shown that TLR2/TLR1 heterocomplex recognized the fibrillar structure of amyloids from both prokaryotic and eukaryotic origin including curli, Aβ and serum amyloid A (SAA) (158, 160–162). In the case of curli, the adaptor molecule CD14 further enhanced the recognition of curli via the TLR2/TLR1 heterocomplex (163). These data instigated further studies investigating whether the conserved fibrillar structure of amyloids serve as a pathological molecular signature for the innate immune system. Consistent with this idea, fibrillar curli (164), Aβ (14), serum amyloid A (165), and IAPP (166) elicited IL-1β cytokine production by directly activating the NLRP3 inflammasome in macrophages. This process impacts the innate immune system in multiple ways: (1) TLR2 activation initiated the pre-IL-1β production and amyloid internalization, (2) NLRP3 inflammasome activation by cytosolic fibrils activated caspase1 and cleaved the pre-IL-1β into mature IL-1β (164). In addition to TLR2, possible activation of TLR4 and TLR6 by Aβ was also reported (167, 168). However, it is not known whether the observed activation of TLR4 and TLR6 was due to the generation of additional Aβ structural conformations during *in vitro* fibrillization or any other contaminating factors. In invertebrates, amyloid formation is key to activation of the innate immune system and host defense. SAA from marine bivalves resembling SAA from vertebrates is a potent acute phase protein and are induced upon bacteria infection (169). In insects such as *Heliothis virescens*, the functional amyloid P102 is synthesized and released to protect against pathogens such as bacteria and parasites. This can occur in response to lipopolysaccharide stimulation (170). The secreted amyloid layer acts as a molecular scaffold to promote localized melanin synthesis and immune cell adhesion to foreign invaders (171). However, it is unknown whether they play a role in direct receptor binding.

Previously, we showed that AMPs like LL37 can self-assemble into an amyloid-like superhelical protofibril to present spatially ordered DNA to TLR9, and that AMP self-assembly with immune ligands can enable signaling through a broad range of PRRs without being direct agonists. Interestingly, nucleic acids have previously been shown to accelerate amyloid fibrillation and serve as molecular templates for self-assembly (172, 173). AD amyloids like Aβ in particular have a propensity to bind to DNA (174) and co-localize within nuclei of affected cells (175, 176). Autoimmune responses to Aβ-containing amyloid structures have been described in AD patients (177). PD-associated α-synuclein fibrils have the ability to self-assemble with DNA (178). Surprisingly, another endogenous amyloid serum amyloid P component (SAP) was shown to be protective against lupus by binding to DNA to prevent formation of anti-DNA antibodies (179, 180), suggesting that perhaps different amyloids are involved in regulating inflammation and recognition of immune ligands. Previously, we showed how structural scaffolding of immune ligands like DNA by AMPs and amyloids dramatically affects immune outcomes (10, 43, 56). AMP-DNA complexes with inter-DNA spacings well-matched with the size of TLR9 amplifies cytokine production, but those with spacings that are much smaller or larger can actually inhibit TLR9 activation and inflammation (4, 43, 45). SAP may potentially regulate inflammation by out-competing binding of proinflammatory amyloids to DNA. This challenges the notion that amyloid assembly is strictly proinflammatory or pathologic.

The ability of amyloids to act as a carrier for nucleic acids to promote endosomal TLR signaling was only recently discovered. Di Domizio et al. showed that artificially formed amyloid fibrils bound to DNA to form amyloid-DNA complexes (181). When administered systemically, these amyloid-DNA promoted systemic autoimmunity, autoantibody production, and lupus-like syndromes in mice by amplifying TLR9 activation in pDCs (6) (Figure 3). A similar observation was made with curli proteins and eDNA found at close proximity in the extracellular matrix of the biofilm. Curli and eDNA formed irreversible complexes together. Similar to what was observed with human amyloids, DNA accelerated the self-assembly process of bacterial amyloid curli (182). Incorporation of DNA into curli rendered DNA resistant to enzymatic degradation. Systemic administration of curli-DNA complexes induced autoantibody production and type I interferon production (12) suggesting that complexes of curli-like bacterial amyloids with DNA may promote inflammatory disorders (183). These findings are fascinating in the setting of our previous work showing that LL37 self-assembles into amyloid-like protofibrils to amplify TLR9 activation. We set out to examine the structures of curli-DNA complexes and found that, similar to LL37 and other AMPs and chemokines, curli was able to organize DNA into geometrically optimal nanostructures to amplify TLR9 activation (Figures 2C,D). Immune activation occurred via a two-step process—curli-DNA complexes were first internalized into immune cells via binding to TLR2 (158–160) and then activated TLR9 once inside the endosome leading to the generation of type I interferons (5). Engagement of TLR2 and TLR9 also contributed to the autoantibody production through unknown mechanisms.

For the longest time, it has been known that infections initiate and/or exacerbate autoimmune diseases. However, the mechanisms of how infections trigger autoimmunity remained a mystery. Besides curli producing enteric bacteria, many important human pathogens such as *Borrelia burgdorferi* (184), *Mycobacterium tuberculosis* (185)*, Pseudomonas aeruginosa* (186, 187), and *Staphylococcus aureus* (188) also produce amyloids. Individuals infected with these pathogens develop some form of autoimmune sequelae such as inflammatory arthritis (13). Phenol soluble modulins (PSMs) from *Staphylococcus* biofilms (189–191) and Fap amyloids from *Pseudomonas* biofilms (186) have been studied concisely, but at present, the mechanisms of DNA binding by other functional amyloids remain unclear, and it remains to be seen whether this has consequences for immune signaling and inflammation. Nevertheless, extracellular DNA is known to facilitate the formation of functional amyloids in *Staphylococcus* biofilms (192), and PSMs are known to bind directly to human formyl peptide receptor 2 (FPR2) (193). Together, these studies strongly suggests a link between chronic bacterial infections, biofilms, and autoimmune diseases (13, 194) (Figure 4). By therapeutically targeting curli amyloid fibers (195), disruption could potentially eradicate bacterial biofilms and secondary autoimmunity.

Formation of amyloid deposits by subunits of different amyloid fibrils is termed as cross-seeding. The co-existence of combinations of α-synuclein, tau, prion protein, and Aβ have all been observed in amyloid deposits in humans (144). In the past several years, few studies also investigated cross-seeding events and a possible link between neurodegenerative diseases and bacterial amyloids. Cross-seeding between SAA and curli was reported in a mouse model of secondary amyloidosis (147). Recent studies have shown that curli can also seed the self-assembly of human α-synuclein (6, 196–198). Colonization of α-synuclein-overexpressing mice with curli-producing *E. coli* exacerbates motor impairment and GI dysfunction, and promotes α-synuclein deposition in the brain (199). However, the spatial interactions between bacterial and host amyloids that would allow for cross-seeding and how these interactions could be influenced by binding to nucleic acids to induce inflammation still remains unknown. We feel that this is an area that should attract and reward attention.

## Conclusions and Outlook

In this review, we discussed the unique functional reciprocity of amyloids and antimicrobial peptides, and how supramolecular self-assembly changes our understanding of their respective roles in host defense and immune activation. We outlined recent work highlighting novel molecular mechanisms for AMP-mediated immunomodulation via TLRs, and implications for antimicrobial responses and inflammatory diseases. We then compared AMP and amyloid self-assembly in the contexts of antimicrobial and membrane-remodeling activity, cytotoxicity, and immune signaling using LL37, Aβ, and curli as fundamental examples.

By critically examining the AMP and amyloid literature together, we discover several convergent themes. First, the amphiphilic properties unique to AMPs and amyloids enable them to cooperatively self-assemble into supramolecular nanostructures to modulate the innate immune system and defend against microbial infections. AMPs, which were thought of as only having antimicrobial function, are now known to modulate innate immune receptors by forming amyloid-like protofibrils and scaffolding canonical immune ligands like DNA and RNA into geometrically organized patterns (Figure 2). Recognition of these complexes by the immune system drives autoimmunity in diseases like lupus, psoriasis, and scleroderma. In a parallel direction, functional bacterial amyloids such as curli from *Salmonella* has shown how these stromal biofilm proteins organize eDNA into cognate spatially ordered complexes to induce autoimmunity in diseases like lupus. Further studies will be required to map out the immune activation landscape of both eukaryotic and prokaryotic amyloids and their distinct mechanisms (Figure 3). For example, exploring how amyloids bind to other immune ligands and identifying the structural rules for immune activation would be incredibly fascinating, analogous to our work with AMP self-assembly. Can we adapt this paradigm to explain autoimmune sequelae of other bacterial infections? We imagine that lessons learned from work on the α-helical AMPs can inform new research directions for α-helical amyloids such as the *Staphylococcus* PSMs, and vice versa. Similarly, our strong understanding of the self-assembly of β-sheet amyloids may inform a better understanding about how β-sheet rich AMPs and AMP-like molecules such as chemokines oligomerize.

Second, the revolutionary work demonstrating that Aβ, which has no known primary function, is an AMP that protects the nervous system against bacterial and fungal infections fundamentally challenges our view of endogenous human amyloids as solely pathologic. This model of Aβ activity suggests that excessive β amyloid deposition in AD and pathogenesis may not necessarily arise from an intrinsic abnormal propensity for Aβ to aggregate, but rather as a consequence of dysregulation of the brain's normal host defense system against invasive infections, similar to how dysregulation of AMP expression and production in tissues can adversely lead to autoimmune diseases (Figure 4). The discovery that α-synuclein, which also has no previous known primary function, is a chemoattractant and is induced to alert the immune system during gut infections opens up incredible opportunities for discovery. Are there other amyloids with hidden antimicrobial activity with potential roles in host defense? What are the primary roles of other endogenous amyloids?

We are just beginning to elucidate the role of supramolecular assembly in immune recognition and modulation. Recent studies have shown that innate immune receptor adaptor proteins like melanoma differentiation-associated protein 5 (MDA5), which senses cytosolic dsRNA, can self-assemble into amyloid-like helical filaments in the presence of dsRNA (200, 201). Helical filament assemblies can also be observed in the signaling pathways of the RIG-I-like receptors (RLRs), AIM2-like receptors (ALRs), and mitochondrial antiviral-signaling protein (MAVS) (202–204). Given that we know how AMPs and amyloids self-assemble with nucleic acids to talk to TLRs, further work will be required to illuminate how they interact with filamentous assemblies of cytoplasmic immune receptors. In summary, we hope that this review will serve to highlight the advances, opportunities, and outlook for the AMP and amyloid communities, and stimulate collaborations between AMP and amyloid biologists, immunologists, as well as bioengineers.

## Author Contributions

All authors listed have made a substantial, direct and intellectual contribution to the work, and approved it for publication.

## Conflict of Interest

The authors declare that the research was conducted in the absence of any commercial or financial relationships that could be construed as a potential conflict of interest.
